# Leveraging Machine Learning Techniques to Forecast Chronic Total Occlusion before Coronary Angiography

**DOI:** 10.3390/jcm11236993

**Published:** 2022-11-26

**Authors:** Yuchen Shi, Ze Zheng, Yanci Liu, Yongxin Wu, Ping Wang, Jinghua Liu

**Affiliations:** 1 Center for Coronary Artery Disease (CCAD), Beijing Anzhen Hospital, Capital Medical University, Beijing Institute of Heart, Lung and Blood Vessel Diseases, 2 Anzhen Road, Chaoyang District, Beijing 100029, China; 2 Zhengzhou Central Hospital Affiliated to Zhengzhou University, Zhengzhou 450007, China

**Keywords:** chronic total occlusion, coronary artery disease, machine learning, prediction

## Abstract

Background: Chronic total occlusion (CTO) remains the most challenging procedure in coronary artery disease (CAD) for interventional cardiology. Although some clinical risk factors for CAD have been identified, there is no personalized prognosis test available to confidently identify patients at high or low risk for CTO CAD. This investigation aimed to use a machine learning algorithm for clinical features from clinical routine to develop a precision medicine tool to predict CTO before CAG. Methods: Data from 1473 CAD patients were obtained, including 1105 in the training cohort and 368 in the testing cohort. The baseline clinical characteristics were collected. Univariate and multivariate logistic regression analyses were conducted to identify independent risk factors that impact the diagnosis of CTO. A CTO predicting model was established and validated based on the independent predictors using a machine learning algorithm. The area under the curve (AUC) was used to evaluate the model. Results: The CTO prediction model was developed with the training cohort using the machine learning algorithm. Eight variables were confirmed as ‘important’: gender (male), neutrophil percentage (NE%), hematocrit (HCT), total cholesterol (TC), high-density lipoprotein cholesterol (HDL), ejection fraction (EF), troponin I (TnI), and N-terminal pro-B-type natriuretic peptide (NT-proBNP). The model achieved good concordance indices of 0.724 and 0.719 in the training and testing cohorts, respectively. Conclusions: An easy-to-use tool to predict CTO in patients with CAD was developed and validated. More research with larger cohorts are warranted to improve the prediction model, which can support clinician decisions on the early discerning CTO in CAD patients.

## 1. Introduction

Chronic total occlusion (CTO) is defined as complete luminal obstruction without antegrade flow for an estimated duration of at least 3 months, according to historical clinical data [1]. It is diagnosed in 16% to 20% of coronary artery disease (CAD) patients undergoing coronary angiography (CAG) [2]. Despite the high prevalence of CTO, CTO recanalization has historically made up a small portion of the total volume of percutaneous coronary intervention (PCI). This is probably because of a combination of factors, including a low success rates, a high incidence of complications, a lengthy procedure, a high costs, and a lack of clinical benefit that is thought to outweigh the costs [3]. Therefore, early recognition and easily measured risk factors of CTO have the greatest potential for early intervention.

In order to improve risk stratification in patients, efforts have been made to develop prognostic predictive tools or risk scores to identify CTO before CAG [4]. Although some previous studies have analyzed potential predictors related to the high incidence rate of CAD and established a relevant model for CAD in patients before CAG, no research has attempted to conduct a rigorous analysis of the CTO data contained within clinical features as a means to produce more personalized CTO risk assessments [5].

In this study, a preoperative CTO risk model based on clinical demographics, echocardiography, and laboratory indexes was constructed by using a machine learning algorithm. This may be observed as an unmet need and an emerging opportunity, as promising computational analysis has demonstrated that it provides clinically valuable predictions in CTO risk and beyond.

## 2. Methods

### 2.1. Study Cohort

In total, 1105 patients with chest pain and suspected CAD who underwent first-time CAG, between January 2019 and December 2019 at Beijing Anzhen Hospital, Capital Medical University, were consecutively enrolled, which formed the training cohort for this study. The patients with CAG or CABG before were excluded. Between January 2020 and December 2020, an independent cohort of 368 consecutive patients was enrolled prospectively with the same inclusion and exclusion criteria. They formed the testing cohort for this study. The entire patients were assigned to the CTO group (*n* = 317) or the non-CTO (*n* = 1156) group based on the procedural outcome. CTO was defined as an occlusion lasting longer than 3 months based on the first occurrence of angina pectoris, previous angiogram, or previous infarction and thrombolysis in myocardial infarction grade 0. Non-CTO was defined as a stenosis of at least 50% of the luminal diameter in at least one branch of the main coronary artery. The CTO was diagnosed independently by two experienced cardiac interventionists (Yongxin Wu and Yanci Liu) and independently checked by another experienced cardiac interventionist (Ze Zheng). Any disagreements between the cardiac interventionists were resolved by discussion with a fourth investigator (Jinghua Liu). The study protocol was approved by the Human Research Ethics Committee of Beijing Anzhen Hospital, Capital Medical University, Beijing, China, and the study adheres to all the principles of the Declaration of Helsinki. The Ethics Committee Approval Code is 2022177X. Given the retrospective study design, informed consent is waived by the Human Research Ethics Committee of the Beijing Anzhen Hospital, Capital Medical University, Beijing, China.

### 2.2. Data Collection

Baseline clinical demographics, laboratory indexes, and angiography characteristics were obtained for each enrolled patient. Age, sex, and medical history information were acquired using questionnaires. Body mass index (BMI) is calculated in kilograms divided by square meters of height. Current tobacco use is defined as the ongoing use of more than one cigarette per day for 6 months. Alcohol consumption is defined as drinkers consuming more than 40 g ethanol (men) or 20 g ethanol (women) per day. Blood pressure in the seated position was measured using an aneroid sphygmomanometer after resting for a minimum of 5 min. The average of three measures was recorded as the final measurement. Hypertension is defined as systolic pressure (SBP) ≥ 140 mmHg, diastolic pressure (DBP) ≥ 90 mmHg, or self-declares the usage of any antihypertensive medication. Diabetes is defined as a fasting plasma glucose level of over 126 mg/dL, a non-fasting plasma glucose level of over 200 mg/dL or self-declares the usage of any antidiabetic medication. Blood samples were taken in a fasting state. White blood cell (WBC), red blood cell (RBC), hemoglobin (Hb), platelet (PLT), hematocrit (HCT), neutrophil (NE), neutrophil percentage (NE%), lymphocyte (LYM), monocyte (MONO), glucose (Glu), HbA1C, triglyceride (TG), total cholesterol (TC), high-density lipoprotein cholesterol (HDL), low-density lipoprotein cholesterol (LDL), free fatty acids (FFA), Non-HDL, sdLDL, homocysteine (Hcy), uric acid (UA), creatinine (Cr), brain natriuretic peptide (BNP), NT-proBNP, high-sensitivity C-reactive protein (hs-CRP), creatine kinase-MB (CK-MB), and troponin I (TnI) were measured from blood samples by standardized laboratory methods. The estimated glomerular filtration rate (eGFR) was calculated in the modified calculated using the CKD-EPI study equation. Angiography characteristics included the number of vessel lesions and the location of the CTO lesion, including the left main coronary artery (LM), left anterior descending branch (LAD), left circumflex artery (LCX), right coronary artery (RCA), and diagonal branch (D1). All angiography characteristics were calculated by two experienced interventional cardiologists.

### 2.3. Statistical Analysis

Data for continuous variables are expressed as mean ± standard deviation, and the difference between groups is examined using Student’s *t*-test. Meanwhile, the categorical variable data is expressed as frequency and percentages, of which the difference between the groups is analyzed using the chi-square test or Fisher’s exact test. 

Univariate logistic regression analysis was performed to identify factors related to CTO prediction. Multivariate logistic regression analysis was performed with the selection of univariate logistic regression analysis, in which the *p*-value levels of the inclusion and exclusion criteria were established as 0.05, respectively. SPSS version 23.0 (SPSS Inc., Chicago, IL, USA) was used to perform statistical analyzes in this study.

### 2.4. Machine Learning Model Construction and External Testing

The R packages with eXtreme Gradient Boosting (XGBoost), XGBoostExplainer, and machine learning in R were used to create predictive models. The training cohort formed by patients who met the inclusion and exclusion criteria between January 2019 and December 2019 at Beijing Anzhen Hospital, Capital Medical University. The testing cohort formed by patients who met the same inclusion and exclusion criteria between January 2020 and December 2020.

The Gini impurity was used to measure the importance of variables, which provided the contribution of the predictive variables to the model [6]. The relative importance score was used to demonstrate the significance of the predictive variables [7]. A receiver operating characteristic curve (ROC) was used to assess the predictive model [8]. Machine learning model construction and external testing were performed using the R software version 4.2.0 (R Foundation for Statistical Computing, Vienna, Austria) [9].

## 3. Results

### 3.1. Baseline Demographics of the Study

The baseline clinical demographics of the study population are shown in Table 1. There were no differences in age, alcohol consumption, BMI, DBP, hypertension, diabetes mellitus, dyslipidemia, prior stroke, and medication history of PPIs between the CTO group and the non-CTO group. Compared to the non-CTO group, CTO group had a higher male rate, current tobacco use rate, left ventricular end diastolic diameter (LVEDD), medication history of clopidogrel, ticagrelor, β-blocker, nitrates, and diuretics. The SBP, ejection fraction (EF), medication history of ACEI (angiotensin converting enzyme inhibitors)/ARB (angiotensin receptor blocker), statin, and CCB were lower in the CTO group than in the non-CTO group.

### 3.2. Baseline Laboratory Indexes of the Study

Compared to non-CTO controls, the CTO group had higher WBC, NE%, MONO, TG, non-HDL, Hcy, UA, Cr, BNP, NT-proBNP, CK-MB, and TnI, but lower PLT and HDL. There were no differences in RBC, Hb, HCT, LYM, Glu, HbA1C, TC, LDL, FFA, sdLDL, eGFR, and hs-CRP. The detailed data and units for all variables are shown in Table 2. 

### 3.3. Baseline Clinical Angiography Characteristics of the Study

Table 3 shows the baseline clinical angiography characteristics of the study. The CTO group had a higher rate of multivessel lesions compared to the non-CTO group. More than half of the patients in the CTO group have 3-vessel lesions. In contrast, nearly half of the patients in the non-CTO group have 1-vessel lesions. The incidence of CTO is RCA, LAD, LCX, and others.

### 3.4. Baseline Clinical Characteristics of the Training and Testing Cohort

Table 4 summarizes the clinical characteristics of the training cohort (*n* = 1105) and the testing cohort (*n* = 368). Clinical demographics that included sex, age, current tobacco use, alcohol consumption, BMI, SBP, and DBP were not significantly different between both groups (all *p >* 0.05). Additionally, the incidences of hypertension, diabetes mellitus, dyslipidemia, and a history of previous stroke did not have significant differences between both groups (all *p >* 0.05). Similarly, there was no statistical significance in echocardiography and laboratory indexes (all *p >* 0.05).

### 3.5. Baseline Clinical Characteristics of the Training Cohort between the CTO and Non-CTO Groups

The baseline clinical characteristics of the training cohort are shown in Table 2. Compared to non-CTO controls, patients with CTO are more likely to be male and smokers. Surprisingly, they have lower SBP and DBP, but the prevalence of hypertension is comparable. They are more frequently encountered with a decreased EF and an increased LVEDD. With baseline laboratory indexes between the two groups, the CTO group demonstrated significantly higher WBC, NE, HbA1C, TG, HDL, Hcy, Cr, BNP, NT-proBNP, and Mb, but lower PLT, LYM%, HDL, and TnI. There were no differences in age, alcohol consumption, BMI, diabetes mellitus, dyslipidemia, prior stroke, RBC, Hb, LYM, MONO, Glu, TC, LDL, FFA, sdLDL, UA, eGFR, and hs-CRP between the two groups.

### 3.6. Univariate Logistic Regression Analysis

Univariate logistic regression analysis was performed to explore the relationship between all clinical characteristics of the patients (Table 5) and the diagnosis of CTO. Nine variables were selected by univariate logistic regression analysis. Table 6 showed the odds ratios (OR), 95% confidence intervals (CI), and *p*-values for each of the variables: gender (OR: 2.929, 95% CI: 1.985–4.323, *p* < 0.0001), NE% (OR: 1.173, 95% CI: 1.086–1.267, *p* < 0.0001), HCT (OR: 0.957, 95% CI: 0.925–0.991, *p* = 0.013), TC (OR: 3.693, 95% CI: 1.14–11.964, *p* = 0.029), HDL (OR: 0.129, 95% CI: 0.036–0.458, *p* = 0.002), EF (OR: 0.965, 95% CI: 0.947–0.984, *p* < 0.0001), TnI (OR: 0.645, 95% CI: 0.484–0.859, *p* = 0.003), CK-MB (OR: 1.002, 95% CI: 1.001–1.003, *p* < 0.0001), and NT-proBNP (OR: 1.000, 95% CI: 1.000–1.001, *p* < 0.0001) were implicated in the risk of CTO patients.

### 3.7. Multivariate Logistic Regression Analysis

Multivariate logistic regression analysis was used to determine the significant independent predictors of CTO. Nine predictors selected by the univariate logistic regression analysis were selected using multivariate logistic regression analysis to determine the independent factors that predict CTO before CAG. Eight variables were included in the final model. As shown in Table 6, gender (OR: 2.860, 95% CI: 1.949–4.197, *p* < 0.0001), NE% (OR: 0.849, 95% CI: 0.787–0.915, *p* < 0.0001), HCT (OR: 1.041, 95% CI: 1.006–1.077, *p* = 0.021), TC (OR: 0.262, 95% CI: 0.081–0.849, *p* = 0.026), HDL (OR: 7.658, 95% CI: 2.158–27.180, *p* = 0.002), EF (OR: 1.036, 95% CI: 1.016–1.056, *p* < 0.0001), TnI (OR: 1.580, 95% CI: 1.185–2.107, *p* = 0.002), and NT-proBNP (OR: 1.000, 95% CI: 0.999–1.000, *p* < 0.0001) were identified as independent factors affecting CTO diagnosis.

### 3.8. Machine Learning Algorithm Model

**Figure 1:**
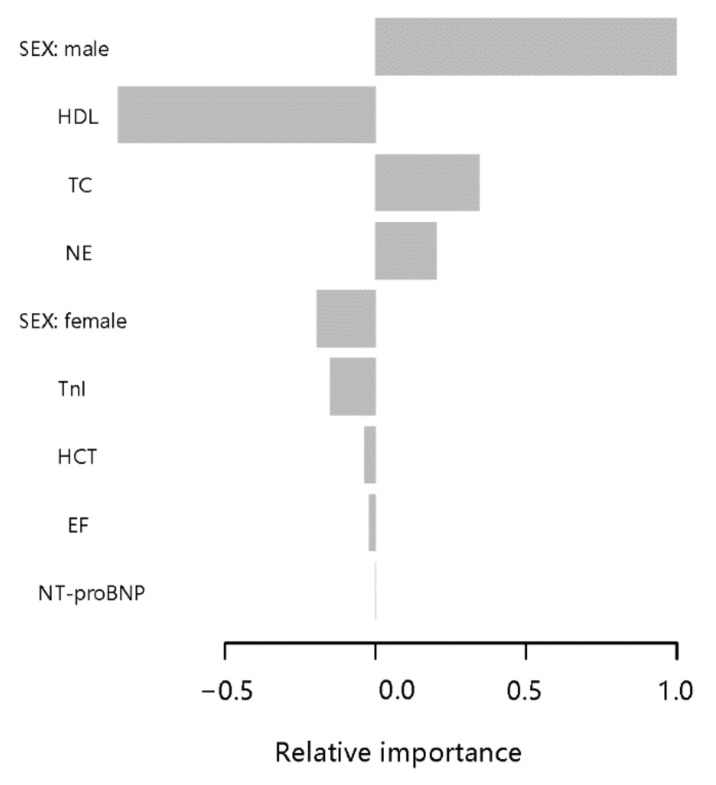
Parameters by predictive value in the extreme gradient boosting (XGBoost) model. To predict CTO, gradient boosting used various variables based on their importance in prediction modeling. In this analysis, the sex and HDL had higher values in the prediction of CTO than other features of patient.

After tuning the XGBoost model, the parameters of the XGBoost model were finally max_depth = 4, subsample = 0.63, colsample_bytree = 0.51. Based on the Gini impurity, the important predictors in the CTO prediction model are summarized in Figure 1. The three aforementioned predictors in the predictive model were male, HDL, and TC. The relative importance score for each is 0.84, −0.71, and 0.29, respectively.

### 3.9. Performance of the Machine Learning Model in the Training and Testing Cohorts

**Figure 2:**
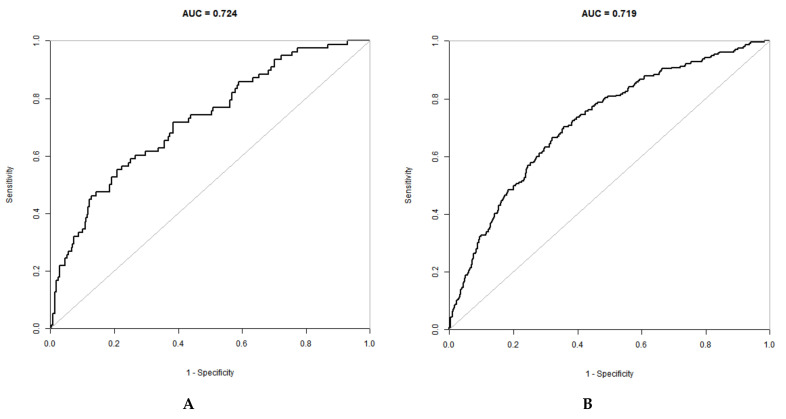
Receiver operating characteristics curve in the training cohort (**A**) and the testing cohort (**B**) for the machine learning model.

To further assess the performance of the models, the ROC curves and the corresponding AUC were evaluated (Figure 2). As Figure 2A showed, the machine learning model achieved an AUC of 0.724 with an accuracy of 0.800 (95% CI, 0.755–0.8398), sensitivity of 80.57%, and specificity of 66.67% in the training cohort. A total of 368 patients were included in the testing cohort between January 2020 and December 2020. In the testing cohort, the AUC was 0.719 with an accuracy of 0.786 (95% CI, 0.761–0.810) (Figure 2B). There was no statistical difference in AUC between training cohort and testing cohort (*p* > 0.05).

### 3.10. The Machine Learning Model Score System for Clinical Utility

**Figure 3:**
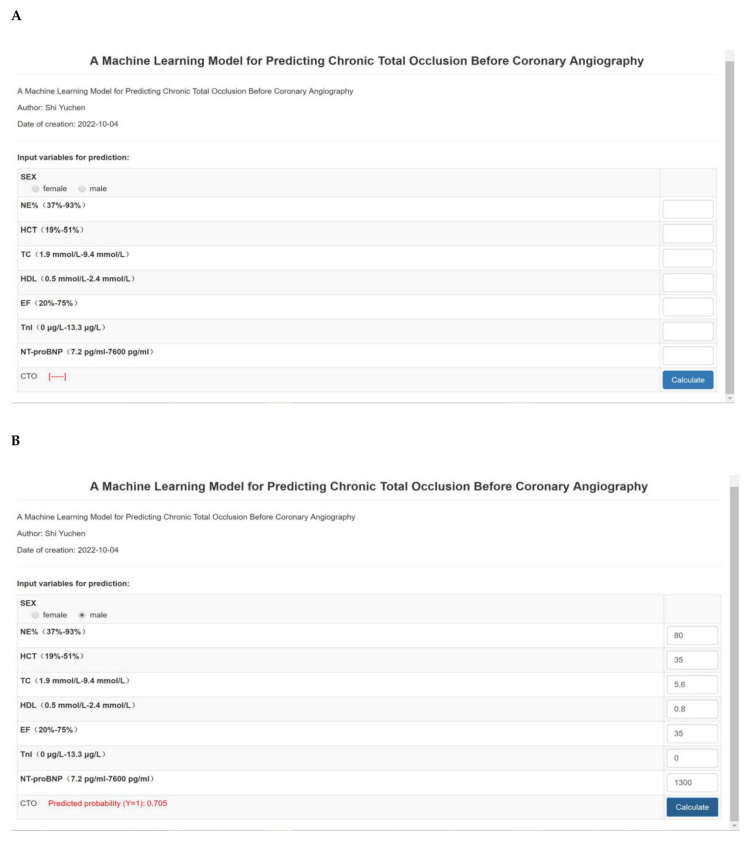
The URLs of the web calculators for our model are as follows: http://www.empowerstats.net/pmodel/?m=29484_CTOBeforeCAG, accessed on 4 October 2022. (**A**) is the flame of the model. (**B**) is an example that if the patient is male with NE% of 80%, HCT of 35%, TC of 5.6 mmol/L, HDL of 0.8 mmol/L, EF of 35%, TnI of 0 μg/L, and NT-proBNP of 1300 pg/mL, the probability of CTO might be 70.5%.

According to the weight coefficient score for each predictor in the model, as Figure 3A showed, we developed the machine learning model scoring system into a web-based calculator (http://www.empowerstats.net/pmodel/?m=29484_CTOBeforeCAG, accessed on 4 October 2022). As an example to better explain the model (Figure 3B), if the patient is male with NE% of 80%, HCT of 35%, TC of 5.6 mmol/L, HDL of 0.8 mmol/L, EF of 35%, TnI of 0 μg/L, and NT-proBNP of 1300 pg/mL, the probability of CTO might be 70.5%.

## 4. Discussion

In this study, we developed a model based on machine learning algorithms to predict CTO patients through only basic patient information such as clinical demographics, echocardiography, and laboratory indexes. We found eight easily captured variables, including gender (male), NE, HCT, TC, HDL, EF, TnI, and NT-proBNP, with relatively accurate prediction ability. To the best of our knowledge, this is the first attempt to develop a predictive model for diagnosing CTO based on machine learning.

Although the event of CAD has been related to varied traditional risk factors, as well as sex (male) and lipid metabolism, their role within the etiology of CTO remains less understood [10]. To date, most research has centered on stable CAD or acute coronary syndrome; there has been no information on risk factors for CTO patients [11]. Additionally, reliable tools for predicting CTO in a timely manner before CAG are lacking. By converting the total score into a continuum of individual scores using a machine learning algorithm, we developed a relatively accurate model to predict the probability of CTO before CAG. Since recanalization of a totally occluded vessel needs a good quantity of time and finance, it would be necessary to construct a model with easy implementation by cardiological residents and staff alike [12]. 

The advent of machine learning methodologies could provide a large amount of information available from databases and encourage the development and testing of better predictive models [13]. Machine learning methods use computational algorithms to identify models in large datasets with multiple variables and can be used to construct predictive models [14]. Machine learning has demonstrated the potential for improving diagnostic accuracy and prognostic outcomes over conventional statistical methods [15]. There are also a number of other advantages to machine learning algorithms over traditional statistical modelling. Machine learning algorithms take into account all potential interactions and do not have pre-defined hypothesis, making them less likely to ignore unexpected predictive variables [16]. Predictive models using machine learning algorithms may thus facilitate the recognition of clinically important risk in patients with multiple marginal risk factors that would otherwise not raise clinical concerns [17]. Furthermore, machine learning algorithms can easily integrate new clinical data to continually update and optimize algorithms with minimal oversight [18].

The primary machine learning model of our study was the XGBoost gradient boosted tree model. XGBoost represents a technical building set of trees in a progressive way on the loss of from weak decision tree base learners [19]. It can learn quickly and efficiently from large amounts of data and its great flexibility makes it possible to learn even from missing data [20]. The XGBoost model had a much higher predictive accuracy compared to the generalized linear model, being able to capture complex associations in the data without requiring explicit high-order interactions and non-linear functions [21]. Using these features, predictive models could be developed from clinical demographics, echocardiography, and laboratory indexes, which are readily accessible and reproducible at admission.

The sex of men and dyslipidemia have previously been linked to the incidence of CTO in patients [22]. Consistent with previous researches, the sex and lipid elements in our machine learning model were significant predictors of CTO. Especially for HDL, it has been proposed that the reduction of HDL could alter the stability of HDL particles, which would disrupt the transport of reverse cholesterol and, at least partially, enhance the progression of CAD to CTO [23]. 

Besides risk factors for CAD, our study revealed that EF and NT-proBNP were independently positively related to CTO prediction. Some CTO lesions develop from an undetected acute myocardial infarction [24]. Acute myocardial ischemia and its early pathological changes often damage left ventricular systolic function, and progress to ischemic cardiomyopathy after 3 months [25]. In contrast, TnI was independently negatively associated with the prediction of CTO. In particular, TnI is a biomarker of acute myocardial ischemia [26]. Given this, the increase in TnI may imply a total occlusion lesion of less than 3 months. Taken together, EF, NT-proBNP, and TnI may predict the probability of CTO in patients with CAD.

In addition to non-modifiable variables (male sex) and traditional risk factors for CAD (TC, HDL, EF, NT-proBNP, and TnI) of the individual patient, blood routine levels including HCT and NE%, were also predictors in the machine learning model. HCT is an indicator that reflects the extent of hemoconcentration [27]. It appears plausible that a higher HCT with increased blood viscosity predicts a higher risk of first incident acute myocardial ischemia than a lower HCT [28]. In contrast, lower HCT may refer to CTO rather than acute total occlusion. For NE%, CTO has long been considered to be a low-grade, subclinical, systemic inflammatory chronic disease [29]. Inflammation is at the forefront in initiating and developing the entire course of CTO [30]. CTO develops from the total luminal obstruction of an artery by a thrombus, with subsequent organization and varying degrees of recanalization; often these events are clinically silent [31]. The process of organizing thrombus coincides with the development of intraluminal microvessels accompanied by inflammatory cells, infiltrating smooth muscle cells, and the deposition of the proteoglycan matrix [32]. In CTOs of all ages, there was a close relationship, both in location and intensity, between cell inflammation and vascular wall neovascularization [33]. NE% is an indicator of inflammatory state. Therefore, it may predict the probability of CTO in CAD patients.

On the other hand, testing is also a significant aspect of predictive model construct, since the performance of regression models is usually much higher in the training cohort than in the testing cohort. In our research, the model was validated in a testing cohort and the results were relatively stable, which could reflect the generalizability of the model to a certain extent. 

Taken together, non-invasive tests have been increasingly used for risk stratification and to facilitate clinical decision making in patients with suspected diseases [34]. Subsequently, machine learning models have been developed in a variety of diseases, which are demonstrated to be more accurate in predicting prognosis than traditional staging systems [35]. The machine learning models may improve the detection rate of CTO. However,, the CAG is the gold standard of CTO. The CAG, including the CCTA, is invasive, with a difficult applicable and expensive for patients. Moreover, the CAG or CCTA are not recommended in routine screening for suspected chest pain populations according to medical guidelines. This model may provide an effective and applicable method for physician and primary care doctors based on the data of routine physical examination to screen suspected chest pain patients in the general population and improve the detection rate of CTO without redundant examination. In addition, the combined use of machine learning and the electronic health records of patients could provide clinicians with a more convenient and quick methods to identify CTO in the busy clinical work. Furthermore, we are not only building a predicting model, but also seek the risk factors for CTO. As mentioned in the above, although the event of CAD has been related to varied traditional risk factors, the risk factors within the etiology of CTO remains less understood. To date, most researches have centered on stable CAD or acute coronary syndrome; there has been no information on risk factors for CTO patients. We seek the risk factors for CTO by machine learning model and find eight easily captured variables, including gender (male), NE, HCT, TC, HDL, EF, TnI, and NT-proBNP might be the risk factors for CTO distinguishing from CAD. If we had knowledge about some important details about the classifiers, we might achieve one result that could be much more effective. For example, when we used the machine learning model, the model already selected the most important features according to the information gain values and then split, with the ranking of the features selected by the model potentially providing us with additional information. By focusing on and controlling those high-risk predictors, we would see a more positive tendency throughout the individual’s entire treatment. Delaying the progression of CAD to CTO would be a tremendous relief for individuals, clinicians, and healthcare systems. It is worth noting that our results of the present study just represent a first step for CTO, and must be validated in a larger cohort in the future. An advantage of machine learning than the conventional statistical method is that it can analyze the data when others use our machine learning model to improve the predicted accuracy of CTO. With more use, the accuracy will improve. In addition, other factors such as genetic predisposition, best medical therapy, sports and activity might also play a role in CTO progress. Expecting larger cohort studies with more factors are validated.

The parameters easily available and reproducible at admission were necessary and quantitative. To our knowledge, this is the pioneering research to investigate the predictability of CTO in patients with CAD and is well performed. These results supported the notion that the machine learning model could better understand the risk factors for CTO and even offered an easily used and automated assistive tool to predict the diagnosis in patients with CTO before CAG. 

## 5. Limitations

There are several limitations in our study. First, we only included patients from the Chinese population that the trained models should be further tested in broader groups of patients to ensure additional generalisation of performance. Second, due to the retrospective design, other randomized controlled trials could fully examine its efficacy. Moreover, our results of the present study just represent a first step for CTO, which must be validated in a larger cohort with more variables such as genetic predisposition, best medical therapy, sports and activity. Finally, there is still room for optimization of this predictive model using other advanced statistical methods, such as the least absolute shrinkage and selection operator (LASSO), which may help develop a more accurate prognostic prediction model.

## 6. Conclusions

In summary, we constructed and validated a relatively accurate machine learning model based on clinical demographics, echocardiography, and laboratory indexes, to help the early prediction of the probability of CTO in patients with CAD. More research with larger cohorts that include more patients and more variables such as genetic predisposition, best medical therapy, sports and activity are warranted to improve the prediction model, which can support clinician decisions on the early discerning CTO in CAD patients. 

## Figures and Tables

**Table 1:** Baseline clinical demographic of study patients.

Variable	Overall	CTO		*p* Value
	(*n* = 1473)	Yes (*n* = 317)	No (*n* = 1156)	
Clinical demographic				
Gender (male)	1049 (71.215%)	266 (83.912%)	783 (67.734%)	0.000
Age (y)	60.289 ± 9.368	59.817 ± 10.146	60.418 ± 9.143	0.513
Current tobacco use	697 (47.318%)	181 (57.098%)	516 (44.637%)	0.00011
Alcohol consumption	479 (32.519%)	109 (34.385%)	370 (32.007%)	0.46356
BMI (kg/m^2^)	25.871 ± 3.232	26.097 ± 3.294	25.809 ± 3.213	0.171
SBP (mmHg)	131.559 ± 16.783	129.281 ± 16.903	132.184 ± 16.702	0.001
DBP (mmHg)	77.969 ± 10.863	77.341 ± 11.18	78.142 ± 10.774	0.121
Hypertension	940 (63.815%)	201 (63.407%)	739 (63.927%)	0.864
Diabetes mellitus	475 (32.247%)	114 (35.962%)	361 (31.228%)	0.110
Dyslipidemia	1163 (78.955%)	244 (76.972%)	919 (79.498%)	0.328
Prior stroke	133 (9.029%)	29 (9.148%)	104 (8.997%)	0.933
Echocardiography				
EF (%)	62.649 ± 7.129	59.953 ± 9.225	63.388 ± 6.241	0.000
LVEDD (mm)	30.914 ± 5.315	32.842 ± 6.748	30.385 ± 4.717	0.000
Medication				
Aspirin	1473 (100%)	317 (100%)	1156 (100%)	-
Clopidogrel	685 (46.504%)	176 (55.521%)	509 (44.031%)	0.000
Ticagrelor	284 (19.280%)	117 (36.907%)	167 (14.446%)	0.000
β-blocker	728 (49.423%)	194 (61.199%)	534 (46.194%)	0.000
ACEI/ARB	478 (32.451%)	85 (26.814%)	393 (33.997%)	0.016
Statin	1406 (95.451%)	296 (93.375%)	1110 (96.021%)	0.045
CCB	414 (28.106%)	67 (21.136%)	347 (30.017%)	0.002
Nitrates	697 (47.318%)	186 (58.675%)	511 (44.204%)	0.000
PPIs	1034 (70.197%)	224 (70.662%)	810 (70.069%)	0.838
Diuretics	249 (16.904%)	86 (27.129%)	163 (14.100%)	0.000

CTO, chronic total occlusion; BMI, body mass index; SBP, systolic blood pressure; DBP, diastolic blood pressure; EF, ejection fraction; LVEDD, left ventricular end diastolic diameter; ACEI, angiotensin converting enzyme inhibitors; ARB, angiotensin receptor blocker; CCB, calcium channel blocker; PPI, proton pump inhibitors.

**Table 2:** Baseline laboratory indexes of study patients.

Variable	Overall	CTO		*p* Value
	(*n* = 1473)	Yes (*n* = 317)	No (*n* = 1156)	
Laboratory indexes				
WBC (10^12^/L)	7.118 ± 2.012	7.634 ± 2.244	6.977 ± 1.921	0.000
RBC (10^12^/L)	4.549 ± 0.517	4.518 ± 0.568	4.558 ± 0.502	0.577
Hb (g/L)	139.586 ± 15.951	138.603 ± 17.036	139.856 ± 15.637	0.556
HCT (%)	40.429 ± 4.317	40.075 ± 4.719	40.526 ± 4.197	0.421
PLT (10^9^/L)	220.14 ± 57.26	214.142 ± 59.138	221.785 ± 56.65	0.020
NE (10^9^/L)	4.659 ± 1.745	5.194 ± 2.069	4.512 ± 1.616	0.000
NE% (%)	64.505 ± 8.859	66.89 ± 9.129	63.851 ± 8.673	0.000
LYM (10^9^/L)	1.905 ± 0.635	1.856 ± 0.641	1.918 ± 0.633	0.174
MONO (10^9^/L)	0.382 ± 0.139	0.4 ± 0.148	0.377 ± 0.136	0.025
Glu (mmol/L)	6.561 ± 2.53	6.737 ± 2.59	6.513 ± 2.512	0.088
HbA1C (%)	6.436 ± 1.215	6.505 ± 1.219	6.417 ± 1.214	0.122
TG (mmol/L)	1.716 ± 1.008	1.821 ± 1.104	1.687 ± 0.979	0.022
TC (mmol/L)	4.15 ± 1.009	4.242 ± 1.119	4.125 ± 0.975	0.162
HDL (mmol/L)	1.117 ± 0.268	1.064 ± 0.25	1.132 ± 0.271	0.000
LDL (mmol/L)	2.427 ± 0.868	2.532 ± 0.981	2.398 ± 0.832	0.062
FFA (mmol/L)	0.481 ± 0.254	0.477 ± 0.248	0.482 ± 0.256	0.813
non-HDL (mmol/L)	3.029 ± 0.972	3.165 ± 1.087	2.992 ± 0.935	0.021
sdLDL (mmol/L)	0.704 ± 0.366	0.721 ± 0.382	0.699 ± 0.362	0.509
Hcy (mmol/L)	15.968 ± 8.752	17.07 ± 8.744	15.666 ± 8.734	0.000
UA (mmol/L)	340.925 ± 86.001	354.897 ± 90.095	337.093 ± 84.481	0.005
Cr (μmol/L)	74.384 ± 18.394	79.224 ± 21.531	73.056 ± 17.21	0.000
eGFR (CKD-EPI)	91.502 ± 14.938	89.917 ± 16.326	91.936 ± 14.511	0.058
BNP (pg/mL)	85.574 ± 166.335	133.319 ± 208.564	72.481 ± 150.208	0.000
NT-proBNP (pg/mL)	306.329 ± 678.67	555.554 ± 950.393	237.986 ± 564.059	0.000
hs-CRP (mg/L)	2.021 ± 2.555	2.233 ± 2.671	1.963 ± 2.521	0.088
CK-MB (U/L)	3.668 ± 12.861	4.085 ± 14.836	3.553 ± 12.269	0.026
TnI (μg/L)	0.221 ± 0.797	0.205 ± 0.74	0.225 ± 0.813	0.001

CTO, chronic total occlusion; WBC, white blood cell; RBC, red blood cell; Hb, hemoglobin; HCT, hematocrit; PLT, platelet; NE, neutrophil; NE%, neutrophil percentage; LYM, lymphocyte; MONO, monocyte; Glu, glucose; TG, triglyceride; TC, total cholesterol; HDL, high-density lipoprotein cholesterol; LDL, low-density lipoprotein cholesterol; FFA, Free Fatty Acids; Hcy, homocysteine; UA, uric acid; Cr, creatinine; BNP, brain natriuretic peptide; NT-proBNP, N-terminal pro-B-type natriuretic peptide; hs-CRP, high-sensitivity C-reactive protein; CK-MB, creatine kinase-MB; TnI, troponin I.

**Table 3:** Baseline clinical angiography characteristics of the study patients.

Variable	Overall	CTO		*p* Value
	(*n* = 1473)	Yes (*n* = 317)	No (*n* = 1156)	
Angiography				
0-vessel	47 (3.191%)	0 (0%)	47 (4.066%)	0.000
1-vessel	592 (40.190%)	41 (12.934%)	551 (47.664%)	0.000
2-vessels	417 (28.310%)	97 (30.599%)	320 (27.682%)	0.307
3-vessels	417 (28.310%)	179 (56.467%)	238 (20.588%)	0.000
CTO-vessel				
LM	-	1 (0.315%)	-	-
LAD	-	119 (37.539%)	-	-
LCX	-	85 (26.814%)	-	-
RCA	-	138 (43.533%)	-	-
D1	-	4 (1.262%)	-	-

CTO, chronic total occlusion; LM, left main coronary artery; LAD, left anterior descending; LCX, left circumflex; RCA, right coronary artery; D1, diagonal branch.

**Table 4:** Baseline clinical characteristics of the training and testing cohort.

Variable	Overall (*n* = 1473)	Training Cohort (*n* = 1105)	Testing Cohort (*n* = 368)	*p* Value
Clinical demographic				
Gender (male)	1049 (71.2%)	795 (71.9%)	254 (69%)	0.283
Age (y)	60.289 ± 9.368	60.386 ± 9.305	59.995 ± 9.560	0.866
Current tobacco use	697 (47.3%)	512 (46.3%)	185 (50.3%)	0.190
Alcohol consumption	479 (32.5%)	370 (33.5%)	109 (29.6%)	0.170
BMI (kg/m^2^)	25.871 ± 3.232	25.829 ± 3.205	25.996 ± 3.311	0.466
SBP (mmHg)	131.559 ± 16.783	131.769 ± 16.817	130.929 ± 16.685	0.302
DBP (mmHg)	77.969 ± 10.863	78.008 ± 10.811	77.853 ± 11.033	0.970
Hypertension	940 (63.8%)	709 (64.2%)	231 (62.8%)	0.631
Diabetes mellitus	475 (32.2%)	358 (32.4%)	117 (31.8%)	0.830
Dyslipidemia	1163 (79%)	877 (79.4%)	286 (77.7%)	0.501
Prior stroke	133 (9%)	93 (8.4%)	40 (10.9%)	0.155
Echocardiography				
EF (%)	62.649 ± 7.129	62.776 ± 7.000	62.269 ± 7.502	0.419
LVEDD (mm)	30.914 ± 5.315	30.881 ± 5.28	31.014 ± 5.425	0.842
Laboratory indexes				
WBC (10^12^/L)	7.118 ± 2.012	7.119 ± 2.044	7.114 ± 1.917	0.620
RBC (10^12^/L)	4.549 ± 0.517	4.547 ± 0.515	4.558 ± 0.522	0.657
Hb (g/L)	139.586 ± 15.951	139.652 ± 15.738	139.389 ± 16.592	0.919
HCT (%)	40.429 ± 4.317	40.438 ± 4.272	40.404 ± 4.456	0.987
PLT (10^9^/L)	220.140 ± 57.260	219.949 ± 56.458	220.712 ± 59.681	0.961
NE (10^9^/L)	4.659 ± 1.745	4.664 ± 1.769	4.644 ± 1.675	0.829
NE% (%)	64.505 ± 8.859	64.587 ± 8.918	64.259 ± 8.686	0.614
LYM (10^9^/L)	1.905 ± 0.635	1.905 ± 0.649	1.904 ± 0.591	0.710
LYM%	0.276 ± 0.08	0.276 ± 0.081	0.277 ± 0.079	0.920
MONO (10^9^/L)	0.382 ± 0.139	0.379 ± 0.138	0.390 ± 0.140	0.247
Glu (mmol/L)	6.561 ± 2.53	6.563 ± 2.625	6.555 ± 2.223	0.333
HbA1C (%)	6.436 ± 1.215	6.412 ± 1.191	6.508 ± 1.286	0.296
TG (mmol/L)	1.716 ± 1.008	1.706 ± 1.026	1.746 ± 0.955	0.080
TC (mmol/L)	4.150 ± 1.009	4.164 ± 1.001	4.109 ± 1.031	0.136
HDL (mmol/L)	1.117 ± 0.268	1.125 ± 0.275	1.095 ± 0.247	0.093
LDL (mmol/L)	2.427 ± 0.868	2.433 ± 0.848	2.407 ± 0.924	0.188
FFA (mmol/L)	0.481 ± 0.254	0.482 ± 0.258	0.478 ± 0.244	0.945
non-HDL (mmol/L)	3.029 ± 0.972	3.036 ± 0.964	3.008 ± 0.998	0.337
sdLDL (mmol/L)	0.704 ± 0.366	0.706 ± 0.37	0.700 ± 0.355	0.926
Hcy (mmol/L)	15.968 ± 8.752	16.155 ± 8.902	15.407 ± 8.273	0.287
UA (mmol/L)	340.925 ± 86.001	340.709 ± 86.453	341.573 ± 84.74	0.946
Cr (μmol/L)	74.384 ± 18.394	74.303 ± 18.476	74.627 ± 18.169	0.754
eGFR (CKD-EPI)	91.502 ± 14.938	91.588 ± 14.859	91.244 ± 15.189	0.757
BNP (pg/mL)	85.574 ± 166.335	82.656 ± 162.868	94.334 ± 176.277	0.189
NT-proBNP (pg/mL)	306.329 ± 678.67	290.398 ± 657.883	354.166 ± 736.455	0.038
hs-CRP (mg/L)	2.021 ± 2.555	2.022 ± 2.544	2.016 ± 2.594	0.877
CK-MB (U/L)	3.668 ± 12.861	3.455 ± 12.251	4.305 ± 14.540	0.111
TnI (μg/L)	0.221 ± 0.797	0.21 ± 0.802	0.253 ± 0.785	0.838

BMI, body mass index; SBP, systolic blood pressure; DBP, diastolic blood pressure; EF, ejection fraction; LVEDD, left ventricular end diastolic diameter; WBC, white blood cell; RBC, red blood cell; Hb, hemoglobin; HCT, hematocrit; PLT, platelet; NE, neutrophil; NE%, neutrophil percentage; LYM, lymphocyte; LYM%, lymphocyte percentage; MONO, monocyte; Glu, glucose; TG, triglyceride; TC, total cholesterol; HDL, high-density lipoprotein cholesterol; LDL, low-density lipoprotein cholesterol; FFA, free fatty acids; Hcy, homocysteine; UA, uric acid; Cr, creatinine; BNP, brain natriuretic peptide; NT-proBNP, N-terminal pro-B-type natriuretic peptide; hs-CRP, high-sensitivity C-reactive protein; CK-MB, creatine kinase-MB; TnI, troponin I.

**Table 5:** Clinical characteristics of the training cohort.

Variable	Overall	CTO		*p* Value
	(*n* = 1105)	Yes (*n* = 235)	No (*n* = 870)	
Clinical demographic				
Gender (male)	795 (71.9%)	201 (85.5%)	594 (68.3%)	0.000
Age (y)	60.386 ± 9.305	60.213 ± 10.198	60.433 ± 9.054	0.838
Current tobacco use	697 (63.1%)	131 (55.7%)	381 (43.8%)	0.001
Alcohol consumption	370 (33.5%)	81 (34.5%)	289 (33.2%)	0.719
BMI (kg/m^2^)	25.829 ± 3.205	26.026 ± 3.218	25.776 ± 3.202	0.288
SBP (mmHg)	131.769 ± 16.817	129.013 ± 17.100	132.514 ± 16.672	0.001
DBP (mmHg)	78.008 ± 10.811	76.796 ± 11.163	78.336 ± 10.697	0.018
Hypertension	709 (64.2%)	150 (63.8%)	559 (64.3%)	0.904
Diabetes mellitus	358 (32.4%)	87 (37.0%)	271 (31.1%)	0.088
Dyslipidemia	877 (79.4%)	184 (78.3%)	693 (79.7%)	0.648
Prior stroke	93 (8.4%)	20 (8.5%)	73 (8.4%)	0.953
Echocardiography				
EF (%)	62.776 ± 7.000	60.209 ± 9.199	63.469 ± 6.099	0.000
LVEDD (mm)	30.881 ± 5.280	32.847 ± 6.604	30.349 ± 4.728	0.000
Laboratory indexes				
WBC (10^12^/L)	7.119 ± 2.044	7.673 ± 2.365	6.97 ± 1.922	0.000
RBC (10^12^/L)	4.547 ± 0.515	4.489 ± 0.565	4.562 ± 0.500	0.288
Hb (g/L)	139.652 ± 15.738	137.8 ± 16.672	140.152 ± 15.448	0.175
PLT (10^9^/L)	219.949 ± 56.458	212.809 ± 57.495	221.878 ± 56.051	0.016
NE (10^9^/L)	4.664 ± 1.769	5.258 ± 2.156	4.504 ± 1.613	0.000
NE% (%)	64.587 ± 8.918	67.363 ± 9.017	63.837 ± 8.747	0.000
LYM (10^9^/L)	1.905 ± 0.649	1.836 ± 0.666	1.924 ± 0.643	0.066
LYM%	0.276 ± 0.081	0.248 ± 0.078	0.283 ± 0.080	0.000
MONO (10^9^/L)	0.379 ± 0.138	0.398 ± 0.150	0.374 ± 0.135	0.074
Glu (mmol/L)	6.563 ± 2.625	6.737 ± 2.688	6.516 ± 2.608	0.114
HbA1C (%)	6.412 ± 1.191	6.515 ± 1.204	6.384 ± 1.186	0.040
TG (mmol/L)	150.971 ± 90.791	164.294 ± 102.854	147.373 ± 86.962	0.007
TC (mmol/L)	4.164 ± 1.001	4.218 ± 1.087	4.149 ± 0.977	0.463
HDL (mmol/L)	1.125 ± 0.275	1.063 ± 0.252	1.141 ± 0.278	0.000
LDL (mmol/L)	2.433 ± 0.848	2.497 ± 0.924	2.416 ± 0.826	0.281
FFA (mmol/L)	0.482 ± 0.258	0.47 ± 0.252	0.486 ± 0.259	0.398
nonHDL (mmol/L)	3.036 ± 0.964	3.138 ± 1.057	3.008 ± 0.936	0.114
sdLDL (mmol/L)	0.706 ± 0.370	0.718 ± 0.379	0.702 ± 0.368	0.642
Hcy (mmol/L)	16.155 ± 8.902	17.386 ± 8.884	15.822 ± 8.882	0.001
UA (mmol/L)	340.709 ± 86.453	350.642 ± 90.796	338.026 ± 85.097	0.115
Cr (μmol/L)	74.303 ± 18.476	79.277 ± 21.542	72.959 ± 17.327	0.000
eGFR (CKD-EPI)	91.588 ± 14.859	89.875 ± 16.55	92.05 ± 14.343	0.089
BNP (pg/mL)	82.656 ± 162.868	128.63 ± 199.296	70.238 ± 149.258	0.000
NT-proBNP (pg/mL)	290.398 ± 657.883	518.068 ± 889.899	228.9 ± 564.628	0.000
hs-CRP (mg/L)	2.022 ± 2.544	2.281 ± 2.708	1.952 ± 2.494	0.096
CK-MB (U/L)	3.455 ± 12.251	3.429 ± 12.317	3.462 ± 12.240	0.076
TnI (μg/L)	0.21 ± 0.802	0.187 ± 0.763	0.216 ± 0.812	0.011

BMI, body mass index; SBP, systolic blood pressure; DBP, diastolic blood pressure; EF, ejection fraction; LVEDD, left ventricular end diastolic diameter; WBC, white blood cell; RBC, red blood cell; Hb, hemoglobin; PLT, platelet; NE, neutrophil; NE%, neutrophil percentage; LYM, lymphocyte; LYM%, lymphocyte percentage; MONO, monocyte; Glu, glucose; TG, triglyceride; TC, total cholesterol; HDL, high-density lipoprotein cholesterol; LDL, low-density lipoprotein cholesterol; FFA, free fatty acids; Hcy, homocysteine; UA, uric acid; Cr, creatinine; BNP, brain natriuretic peptide; NT-proBNP, N-terminal pro-B-type natriuretic peptide; hs-CRP, high-sensitivity C-reactive protein; CK-MB, creatine kinase-MB; TnI, troponin I.

**Table 6:** Univariate and multivariate logistic regression analyzes for CTO predictors.

	Univariate Logistic Regression			Multivariate Logistic Regression		
Variables	OR	95%CI	*p*	OR	95%CI	*p*
Gender (male)	2.929	1.985–4.323	0.000	2.860	1.949–4.197	0.000
NE% (%)	1.173	1.086–1.267	0.000	0.849	0.787–0.915	0.000
HCT (%)	0.957	0.925–0.991	0.013	1.041	1.006–1.077	0.021
TC (mmol/L)	3.693	1.14–11.964	0.029	0.262	0.081–0.849	0.026
HDL (mmol/L)	0.129	0.036–0.458	0.002	7.658	2.158–27.180	0.002
EF (%)	0.965	0.947–0.984	0.000	1.036	1.016–1.056	0.000
TnI (μg/L)	0.645	0.484–0.859	0.003	1.580	1.185–2.107	0.002
CK-MB (U/L)	1.002	1.001–1.003	0.000	0.998	0.997–1.002	0.064
NT-proBNP (pg/mL)	1.000	1.000–1.001	0.000	1.000	0.999–1.000	0.000

OR, odds ratios; CI, confidence intervals; NE%, neutrophil percentage; HCT, hematocrit; TC, total cholesterol; HDL, high-density lipoprotein cholesterol; EF, ejection fraction; TnI, troponin I; CK-MB, creatine kinase-MB; NT-proBNP, N-terminal pro-B-type natriuretic peptide.

## Data Availability

The data sets used during the study are available from the corresponding author on reasonable request.
